# Characterizing and ranking computed metabolic engineering strategies

**DOI:** 10.1093/bioinformatics/bty1065

**Published:** 2019-01-12

**Authors:** Philipp Schneider, Steffen Klamt

**Affiliations:** Max Planck Institute for Dynamics of Complex Technical Systems, Analysis and Redesign of Biological Networks, Magdeburg, Germany

## Abstract

**Motivation:**

The computer-aided design of metabolic intervention strategies has become a key component of an integrated metabolic engineering approach and a broad range of methods and algorithms has been developed for this task. Many of these algorithms enforce coupling of growth with product synthesis and may return thousands of possible intervention strategies from which the most suitable strategy must then be selected

**Results:**

This work focuses on how to evaluate and rank, in a meaningful way, a given pool of computed metabolic engineering strategies for growth-coupled product synthesis. Apart from straightforward criteria, such as a preferably small number of necessary interventions, a reasonable growth rate and a high product yield, we present several new criteria useful to pick the most suitable intervention strategy. Among others, we investigate the robustness of the intervention strategies by searching for metabolites that may disrupt growth coupling when accumulated or secreted and by checking whether the interventions interrupt pathways at their origin (preferable) or at downstream steps. We also assess thermodynamic properties of the pathway(s) favored by the intervention strategy. Furthermore, strategies that have a significant overlap with alternative solutions are ranked higher because they provide flexibility in implementation. We also introduce the notion of equivalence classes for grouping intervention strategies with identical solution spaces. Our ranking procedure involves in total ten criteria and we demonstrate its applicability by assessing knockout-based intervention strategies computed in a genome-scale model of *E.coli* for the growth-coupled synthesis of l-methionine and of the heterologous product 1,4-butanediol.

**Availability and implementation:**

The MATLAB scripts that were used to characterize and rank the example intervention strategies are available at http://www2.mpi-magdeburg.mpg.de/projects/cna/etcdownloads.html.

**Supplementary information:**

Supplementary data are available at *Bioinformatics* online.

## 1 Introduction

Bio-based production processes with renewable feedstocks hold a great potential for the sustainable provision of chemicals. Metabolic engineering aims for establishing and improving bioprocesses by redesigning and optimizing the metabolism of microorganisms through genetic and regulatory interventions (Keasling, 2010). There are many examples of successful implementations of metabolic engineering strategies for the bio-based synthesis of a large variety of compounds including platform chemicals, biofuels, amino acids and precursors for bioplastics (Becker and Wittmann, 2015; Choi *et al.*, 2015; Keasling, 2010; Lee and Kim, 2015; Tsuge *et al.*, 2016). Many experimental, but also mathematical tools have been developed to design new cell factories. In particular, genome-scale metabolic models are now available for numerous production hosts (King *et al.*, 2016) and allow the in-depth analysis of metabolic networks and their capabilities by means of constraint-based modeling (Bordbar *et al.*, 2014; Feist *et al.*, 2009; Klamt *et al.*, 2014; Lewis *et al.*, 2012).

Apart from their descriptive role, genome-scale models also support the identification of metabolic intervention strategies. In general, these strategies consist of gene or reaction deletions, insertions or up- and downregulations and, in case of high-volume chemicals, often aim for establishing a stoichiometric coupling of cell growth with the formation of the target product (Long *et al.*, 2015; Machado *et al.*, 2016; Maia *et al.*, 2016; Maranas and Zomorrodi, 2016; von Kamp and Klamt, 2017). With growth-coupled strain designs, the cell must either form the product when it approaches its maximum growth rate (weak coupling) or synthesis of the product is mandatory at any growth rate (strong coupling) (Klamt and Mahadevan, 2015). Several algorithms have been developed to compute intervention strategies that enforce growth coupling. including the OptKnock (Burgard *et al.*, 2003) algorithm and its numerous variations which rely on bi-level optimization (Long *et al.*, 2015; Machado *et al.*, 2016; Maia *et al.*, 2016; Maranas and Zomorrodi, 2016; Tepper and Shlomi, 2010). Another approach is the framework of minimal cut sets (MCSs), which, among other applications, can also be used to enumerate intervention strategies for weak or strong coupling (Hädicke and Klamt, 2011; Klamt, 2006; Mahadevan *et al.*, 2015; von Kamp and Klamt, 2014). Many of the above mentioned methods may generate a large pool of alternative intervention strategies to meet the predefined goals (Kim and Reed, 2010; Maranas and Zomorrodi, 2016; Patil *et al.*, 2005; von Kamp and Klamt, 2014). A necessary step between model-driven design and experimental implementation is therefore an extensive screening and assessment of the proposed strategies to identify the best candidate which has preferably low experimental effort and leads with high probability to a mutant strain with strong performance.

**Fig. 1.**
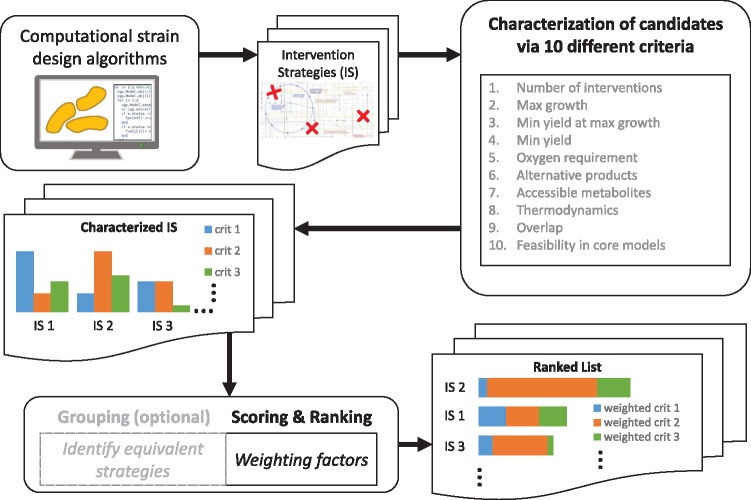
Overview of the proposed characterization criteria and ranking procedure for metabolic intervention strategies

While several approaches have been developed for selecting endogenous and heterologous product synthesis pathways for metabolic engineering (for a review see Wang *et al.*, 2017), we found only a single study (Hartmann *et al.*, 2017) that addressed the problem of a systematic characterization and ranking of intervention strategies (ISs). In this work, we present a catalogue of ten criteria to characterize growth-coupled ISs. Several of these criteria are new and go beyond standard metrics. The presented criteria can be used, in a first step, to preselect certain strategies if some properties are essential or imply exclusion. We then propose a scoring scheme to rank all remaining strategies facilitating a final selection for experimental implementation (Fig. 1) We illustrate our criteria and the ranking scheme by two different case studies where we computed ISs in a genome-scale model of *E.coli* for the growth-coupled production of the amino acid l-methionine and of the heterologous product 1,4-butanediol. In these case studies, the respective pools of ISs were computed as minimal cut sets but the presented criteria and ranking scheme could as well be applied to outputs of other algorithms.

## 2 Materials and methods

We assume that a set of intervention strategies (ISs) has been computed by an appropriate strain design algorithm based on a constraint-based stoichiometric model of the metabolism of the respective wild-type production organism. In constraint-based metabolic models it is assumed that the intracellular metabolites are in steady state leading to the metabolite balancing equation:

Nr=0

where N is the stoichiometric matrix and **r** the vector of net reaction rates. In addition, flux capacity constraints for the reaction rates can be considered:

$${lb}_{i}\leq r_{i}\leq {ub}_{i}$$

In particular, *r_i_* ≥ 0 must be fulfilled for irreversible reactions. Further linear (in)equalities can be added for including other known (e.g. proteome allocation) constraints. The resulting solution space of steady-state flux distributions can be analyzed with a variety of methods (Lewis *et al.*, 2012). The characterization of many properties of ISs is based on analyzing the solution space of the mutant model which is obtained from the wild-type model after implementing the respective interventions (e.g. by setting *lb_i_* and *ub_i_*_._ to 0 if reaction *i* is knocked-out). Dependent on the chosen method, ISs often contain reaction or gene knockouts, which narrow down the solution space, but they may also comprise flux up- and downregulations or the insertion of heterologous metabolic reactions and pathways. The properties listed below evaluate a given IS with respect to its required experimental effort, performance and robustness.

Property 1: Number of required interventions (*#int*)

The number of required interventions is an obvious measure for the future experimental effort and should be preferably small. Nevertheless, the importance of this measure compared to the other criteria may vary and depends on the time and means available for implementation.

Property 2: Maximum growth rate (*μ_max_*)

The maximum growth rate is a measure of a mutant’s viability. In most strain design methods the lower limit for the maximum growth rate (or biomass yield) is defined as a constraint for computing the ISs to guarantee acceptable growth rates. The importance of the maximum growth rate depends on the chosen process type and process parameters. Higher growth rates of the mutant can be a driver for good volumetric productivity, especially in one-step batch fermentation with growth-coupled product synthesis. Combined with the minimum product yield, the maximum growth rate can be used to make statements about the productivity, either through a process simulation (Klamt *et al.*, 2018a; Zhuang *et al.*, 2013) or the determination of related productivity benchmarks, such as the substrate-specific-productivity (SSP) (Feist *et al.*, 2010).

Property 3: Minimum product yield at maximum growth rate ($Y_{P/S}^{min}@{µ}_{max}$)

For strain design algorithms focusing on weak coupling (such as OptKnock or RobustKnock), the product synthesis rate or product yield at maximum growth rate is of major importance since this is considered as the operating point of the mutant after adaptive evolution (Conrad *et al.*, 2011). In the following, we focus on the product yield at maximum growth rate as a performance measure although the product synthesis rate could be used as well. The product yield at the maximum growth rate can be a unique value or lie in a certain range where we then consider the minimum guaranteed product yield as relevant measure. This value can be obtained through two subsequent simple optimizations, namely a maximization of the growth rate followed by a minimization of the product yield under fixed maximum growth rate. In practice, the minimum product yield can be obtained through a technique similar to flux balance analysis (FBA), however, the objective function contains a ratio of the product synthesis and substrate uptake rate and is thus not linear as in FBA. The resulting linear-fractional program (LFP) can be transformed to a linear program and solved to obtain the minimum yield ratio between two fluxes (Klamt *et al.*, 2018b).

Property 4: Minimum product yield ($Y_{P/S}^{min}$)

Sometimes mutant strains may not attain the flux distribution with maximum growth rate in experiments, e.g. due to possible regulatory or pathway capacity constraints. We therefore consider the minimum product yield enforced by an intervention strategy as another criterion. In fact, some strain design algorithms even enforce a minimum product yield for all feasible flux vectors in the mutant, even at non-optimal growth (strong coupling). This criterion thus quantifies the strength of the coupling. Again the minimum product yield can be computed by linear-fractional programming as explained above.

Property 5: Requirement of anaerobic conditions (*O_2_*)

Another criterion for evaluating an IS and the resulting process concerns the necessity of oxygen supply for the mutant strain. Anaerobic growth regimes are often easier to implement in large-scale and then preferred. In fact, some ISs even demand anaerobiosis while others require oxygen. Furthermore, the preference for an aerobic or anaerobic process may also depend on pathway capacities and regulation (van Heerden and Nicol, 2013), product inhibition, process stability and other factors.

Property **6:** Number of alternative products that could disrupt coupling if secreted (*#alterProd*)

Growth-coupled ISs are sometimes not successful in practice because of unexpected by-product secretion abrogating the stoichiometric coupling of growth and product synthesis. To quantify the robustness of a growth-coupled design, we determine the number of metabolites (alternative products) that would lead to a disruption of the coupled growth and product synthesis if the cell was able to secrete or, at least temporarily, to accumulate them. The importance of this approach arises from the fact that there is a large number of promiscuous transporters with varying specificity. The citrate transporter *CitT* is such an example (Pos *et al.*, 1998). It mainly functions as citrate/succinate-antiporter but also shows an affinity towards other C4-dicarboxylates, such as fumarate or aspartate. However, established metabolic models often neglect secondary functionalities of transporters. For the wild type scenario and under most fermentation conditions, this assumption is realistic. Yet, in scenarios where other C4-dicarboxylates are accumulated, its export is possible (Engel *et al.*, 1992) and there have been metabolic engineering approaches for fumarate production, that rely completely on the native *E.coli* C4-dicarboxylate transport systems (Song *et al.*, 2013), showing that the secretion is possible.

In order to decrease the number of necessary interventions and because usually only few metabolites are excreted under a given condition, the set of potential product sinks can initially be reduced to the main fermentation products (von Kamp and Klamt, 2017). Searching for alternative products may then help to identify potential leaks that could abrogate coupling.

**Fig. 2.**
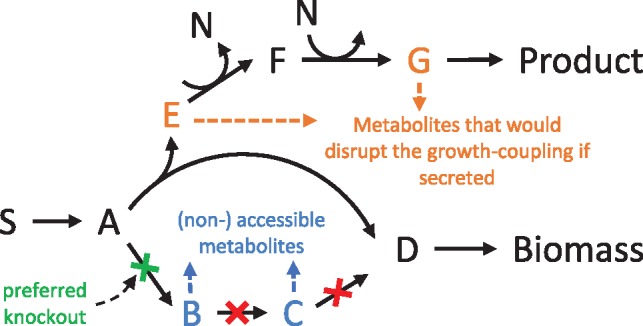
Example for disruption of growth coupling and for accessible metabolites. In the lower branch there are three different cuts (green, blue, blue) that establish coupling of biomass and product synthesis. All of them cut the same pathway and lead to the same solution space but they imply a different number of accessible metabolites. The green cut at the beginning of the pathway is preferred and has the lowest number of accessible metabolites. Choosing the green cut (or, less favored, one of the two red cuts), coupling is ensured and the product is synthesized if the cell grows, however, excretion of the metabolites E and G (but not F) would disrupt growth coupling (Color version of this figure is available at *Bioinformatics* online.)

To test whether the excretion of a given metabolite could potentially disrupt growth coupling, the model is temporarily extended by an export reaction for this metabolite and with FBA it is verified whether the desired growth coupling is then still existent with desired specifications, e.g. with minimum product yield or production rate. This is done, one by one, for all metabolites that do not yet have an exchange reaction. The total number of metabolites that remove the growth coupling with the actual target product gives a measure for the robustness of the IS. An example is illustrated in Figure 2. Using the procedure described above reveals that the secretion of metabolites E or G (orange) would disrupt strong coupling while F is not a possible alternative product because the co-factor N could then not be balanced by the cell.

Mutants that hold less alternative products tend to have a growth coupling that is more robust, for example, because the coupling mechanisms, such as cofactor regeneration, occur at the end of the product synthesis pathway. Our approach also allows one to exclude ISs with by-products that are likely to occur or have already been observed in previous experiments. Sometimes only the combined secretion of multiple alternative products leads to a disruption of growth coupling, however, we do not consider those combinations herein because they are less likely and their detection requires higher computational effort. Furthermore, if the likelihood of secretion can somehow be quantified, the approach could also be extended by associating individual penalty scores for each metabolite. For example, metabolites that are phosphorylated or bound to Coenzyme A are less likely to be secreted.

A useful extension for the described approach is the identification of the nature of the respective coupling strategy. For this purpose, artificial reactions that recover co-factors such as ATP/ADP, NADH/NAD or NADPH/NADP ‘for free’ from the respective unphosphorylated (ADP)/phosphorylated (ADP) or oxidized (NAD(P))/reduced (NAD(P)H) form. If growth coupling is, for example, disrupted by the integration of an NADH oxidizing reaction, this would indicate that the underlying coupling mechanism relies on the balance of reduction equivalents.

Equivalence classes of intervention strategies

In practice, different ISs can lead to identical solution spaces. The reason is the nature of the steady-state assumption. For example, a linear pathway can be interrupted through a cut of any of the sequential reactions. An example is given in Figure 2 where the pathway from A to D can be interrupted by three different cuts. All three cuts (green and two blue cuts) interrupt this pathway and yet lead to the same solution space. Using this criterion, a pool of ISs can always be partitioned into (*equivalence*) *classes* where all ISs of one class have identical solution spaces. To identify equivalence classes, a flux variability analysis is performed for each IS. All ISs with identical flux ranges belong to the same class. It is reasonable to consider only one representative strategy for each class to avoid ranking of redundant solutions, however, it remains to be specified what the best strategy of each class is. This is related to Property 7.

Property 7: Number of accessible metabolites (*#accessMet*)

Regarding linear pathways, an interruption at an intermediate step or at an endpoint of the pathway (blue cuts in Fig. 2) can lead to an undesired accumulation of intermediate metabolites. Therefore, it is advantageous to interrupt pathways at their branching point (green cut in Fig. 2) and thus to minimize the total number of metabolites that can be produced. We therefore propose the number of *accessible metabolites* as a criterion to assess the risk of accumulation (and possible secretion) of metabolites in a given strain design. We suggest to identify the (best) representative for each IS class as the IS with lowest number of accessible metabolites. Accessible metabolites are all those metabolites that can, in principle, be synthesized by the reactions of a metabolic network. Note that this also includes metabolites for which, e.g. due to certain proposed interventions, no further metabolization pathways or sinks (excretion reaction) exist in a redesigned network. In Figure 2, the three different knockouts lead to the same solution space, yet, the number of accessible metabolites differs for each of them and the strategy with the smallest number (the green cut) holds the lowest risk for the accumulation of an intermediate product because the undesired pathway is interrupted at its root. We hence would take the green cut as the representative strategy for this class. In the rare case that multiple strategies of a equivalence class have the same minimal number of accessible metabolites, one may consider only one representative selected by additional criteria (see case study below) or all of them for further evaluation.

To test whether an intracellular metabolite is accessible in a mutant, the model is extended by a sink reaction for this metabolite. The steady-state constraint Nr=0 is then replaced with the weaker constraint Nr≥0, hence, all metabolites are allowed to accumulate. This ensures that metabolites are classified as accessible also if their synthesis requires simultaneous accumulation or excretion of other metabolites. An optimization maximizing the flux through the new sink reaction is then performed to see whether this flux can become non-zero, indicating that the metabolite is accessible. This check is done successively for all metabolites delivering the total number of metabolites that are accessible.

As suggested by Trinh *et al.*, 2015, the undesired interruption of reactions at downstream positions of linear pathways could already be avoided during IS computation. This would not only reduce the number of relevant strategies but also speed up the computation. However, sometimes it is not ad hoc possible to define beginning and end of the respective product synthesis pathway(s), as this may depend on the chosen intervention strategies.

Property 8: Thermodynamics: Optimal max-min driving force (*OptMDF*)

Thermodynamic pathway analysis has been used before in other studies as a feasibility constraint for pathway prediction (Campodonico *et al.*, 2014) and as a criterion for pathway ranking (Carbonell *et al.*, 2014; Kuwahara *et al.*, 2016). The metric of Max-min driving force (MDF) introduced by Noor *et al.* (2014) is an optimization-based technique for determining the maximum thermodynamic driving force and the thermodynamic feasibility of a given metabolic pathway under best-case conditions. The concept of MDF was already successfully applied to genome-scale networks to rank and discriminate biosynthetic pathway candidates for expanding metabolic networks (Asplund-Samuelsson *et al.*, 2018). However, MDF in its original form can only be used to test thermodynamic feasibility of one given pathway. We therefore use the OptMDFpathway method, recently introduced in Hädicke *et al.* (2018), to find the flux vector with growth-coupled product synthesis that yields the maximum MDF (OptMDF) in the entire solution space of the mutant. This quantity will be used as a ranking criterion for the respective intervention strategy. In addition, if the computed OptMDF is smaller than zero, then the corresponding strategy leads to a mutant without any thermodynamically feasible flux distribution and can thus be removed from the pool.

Property 9: Overlap with other intervention strategies (*overlap*)

Regarding experimental implementation it is advantageous to lower the initial risk of failure by choosing one with many fallback options. To have a related measure that correlates with the number of fallback options we quantify the overlap of an IS with others. We first count the number of occurrences of a certain intervention in the entire pool of ISs. The overlap measure for each strategy is then calculated as the sum of the occurrences of all its interventions divided by the number of interventions. A high overlap measure indicates more potential fallback options, which is especially useful when an iterative step-by-step approach is followed in the experimental implementation of the strain design (Harder *et al.*, 2016). When an IS is picked, the first interventions to be implemented would be those with the highest scores (many overlaps), moving successively towards the lower scores (few overlaps), so that an alternative strategy could be used if a strategy change is be required (e.g. due to failure of growth).

Property 10: Feasibility in reduced models (*core-model*)

The underlying mechanisms of different growth-coupled ISs can sometimes be hard to identify. Some recurring patterns are cofactor regeneration, proton balancing, carbon branching and interruption alternative pathways (Alter *et al.*, 2018). Nevertheless, the metabolic routes that are finally used for the synthesis of the product of interest are diverse and, in a genome-scale model, may not be limited to the central carbon metabolism and also comprise pathways with presumably smaller capacities. In principle, constraint-based models could be adjusted to consider known maximum pathway capacities to avoid solutions that rely on high metabolic fluxes through secondary pathways. However, bounds of internal fluxes are usually not known. Furthermore, one may still be interested in solutions with low capacities when there is the prospect of increasing the pathway capacity, e.g. by amplifying the corresponding genes.

We therefore suggest to evaluate the reliance of an IS on pathways with small capacities. For this purpose, we test the computed ISs in a reduced core network that only comprises the major catabolic and anabolic pathways. Alternatively, when a core model is not available, one may choose other criteria, e.g. relying on GO (gene ontology) terms, to mark certain reactions to have only limited capacity. These reactions should then not be essential in a designed strain.

In our examples we used *EColiCore2*, a model of *E.coli’*s central metabolism derived from the genome-scale model *i*JO1366 (Hädicke and Klamt, 2017). It comprises only 20% of the reactions of the full model but reproduces major phenotypes. As the *EColiCore2* is a sub-network of *i*JO1366, we can easily test whether a knockout-based IS, computed in the full model, is also applicable in the smaller model: All interventions that target elements contained in the core model are implemented in the latter and FBA is then used to check whether the desired constraints of the mutant (e.g. minimum product yield when growing) are still fulfilled. If this is not the case, the IS is classified as reliant on secondary pathways and will thus receive a lower score in the ranking procedure.

Scoring and ranking

The ten criteria described above characterize the different ISs and can be used to compare them with each other; first on the basis of each single criterion. To obtain a comprehensive quantitative measure $S_{i}$ for each intervention strategy i, we suggest the calculation of an overall score from the individual scores $S_{i,j}\;$for each of the ten criteria j=1…10. Each criterion score $S_{i,j}$ can take normalized values between 0 and 1. The intervention strategy i that takes on the most unfavorable value on the criterion j attains the score $S_{i,j}=0$ and the strategy *k* with the most favorable value is scored with $S_{k,j}=1$. Concretely, if a high value $U_{i,j}$ of a specified criterion j is desirable [e.g. minimum product yield or maximum thermodynamic driving force (OptMDF)], the score for the strategy i is determined by:

$$S_{i,j}=\frac{U_{i,j}-U_{j,min}}{U_{j,max}-U_{j,min}}.$$

In the case of a preferably low value $U_{i,j}$, the term is:

$$S_{i,j}=\frac{U_{j,max}-U_{i,j}}{U_{j,max}-U_{j,min}}.$$

Criteria with a preferably low value are the number of necessary interventions, the number of metabolites that disrupt the growth-product coupling and the number of accessible metabolites. The score for the aeration requirement $S_{O_{2}}$ takes the value 1 for the anaerobic and 0 for the aerobic case. The score for the feasibility in reduced networks takes the value 1 if the strategy is feasible and 0 if it is infeasible in a reduced network. In the case that an OptMDF value is negative, indicating thermodynamic infeasibility of the IS with given constraints, the individual score as well as the lower reference value ($U_{j,min}$) for the normalization are then set to zero.

The total score $S_{i}$ for an intervention strategy i is then a weighted sum of its scores for the ten criteria:

$$S_{i}=\sum_{j}c_{j}S_{i,j}.$$

The weights $c_{j}\;$can be adapted to reflect (i) the particular relevance of each criterion in a given application and (ii) the spread of the values. A smaller weight should be used for a criterion if its values are distributed over a very narrow range only, indicating low variability. In the simplest and uniform approach one may set all weights to 1. The computed overall scores $S_{i}\;$can finally be used to rank the ISs.

## 3 Results

### 3.1 Case study: model and computation of minimal cut sets

To illustrate our approach, we computed growth-coupled strain designs for two different products: l-methionine and 1,4-butanediol produced via a homologous and a heterologous pathway respectively. We used the *E.coli* genome-scale model *i*JO1366 (Orth *et al.*, 2011) with minor modifications (see supplements) and minimal media with glucose as the sole substrate. The ISs in these examples are knockout-based and computed as minimal cut sets (MCSs) by a variant of the algorithm used by von Kamp and Klamt (2017). Undesired phenotypes that should be eliminated through the MCSs (so-called target flux vectors) were defined as flux vectors with a product yield lower than 30% of the theoretical maximum yield of the respective product. For the protected (desired) phenotypes we assumed a minimum product yield above this 30% threshold and a lower limit of the maximum growth rate of 0.05 h^−1^. To speed up MCS computation, the genome-scale model was first compressed by merging sets of fully coupled reactions and by removing conservation relations (von Kamp and Klamt, 2017). For the MCS search we set an upper limit of 13 reaction knockouts per strategy and the calculation was aborted if (i) the solver finished the search, (ii) a time limit of 24 h was exceeded) or (iii) when 200 MCSs were found in the compressed model. For all computations we used API functions of *CellNetAnalyzer* 2018.1 (Klamt *et al.*, 2007; von Kamp *et al.*, 2017) in MATLAB 2016a with IBM CPLEX 12.6.3 as MILP solver. The MCS computation algorithm was run on an HPC cluster, using 12 CPU slots per computation (2x Intel Xeon X5650–6 cores each) with 28 GB of memory. Standard Gibbs energies $\Delta_{r}G^{'0}$ for computing the maximum MDF in the mutants (OptMDF; property 8) were available for 744 reactions and taken from Hädicke *et al.* (2018). For assessing property 10, the feasibility in reduced models, we used the *EColiCore2* model (Hädicke and Klamt, 2017). The full set of computed MCSs and their properties and ranking can be found in the Supplements.

### 3.2 Preselection and ranking

The ranking of the computed ISs involved two steps. First, to avoid occurrence of many equivalent strategies in the final ranking tableau, a preselection was performed based on equivalence classes of MCSs (see property 7 in Section 2). All MCSs of one equivalence class lead to an identical solution space of steady-state flux vectors when applying the interventions in the model. To identify equivalence classes, we performed for each IS a flux variability analysis and grouped all MCSs with identical flux ranges in one class. We then selected one (the best) representative of each class which has the minimum number of accessible metabolites. If there are, within one class, several MCSs with a minimum number of accessible species, then, from these MCSs, the one with the highestoverall score is selected as the representative.

After preselection, the MCS class representatives underwent the scoring and ranking procedure as described in Section 2. We used the weighting coefficients $c_{\#int}=1.5$, $c_{\mu_{max}}=1$, $c_{Y_{P/S}^{min}@{µ}_{max}}=1$, $c_{Y_{P/S}^{min}}=1$, $c_{O_{2}}=0.5$, $c_{\#alterProducts}=0.5$, $c_{\#accessMet}=0$, $c_{optMDF}=0.5$, $c_{overlap}=0.5$, $c_{core-model}=0.25$. We thus chose slightly larger weights for the first four criteria to emphasize the number of interventions, the maximal growth rate and the minimum product yield during ranking. Note that the number of accessible metabolites is only used (and only reasonable) as criterion for the preselection of ISs in the equivalence classes (see Section 2) and the weight of this score is therefore set to zero in the ranking of the representatives.

### 3.3 Example 1: l-Methionine

Regarding the global market size of the different amino acids, methionine holds the third place after glutamate and lysine. Main applications of methionine lie in livestock (feed additive, especially poultry farming), pharmaceutics and nutrition (Huang *et al.*, 2017; Willke, 2014). Despite many attempts, there are only few successful examples of metabolic engineering approaches that lead to an established industrial bioprocess. In terms of strain design, one of the main hurdles lies in the complex regulation of the l-Methionine biosynthesis in bacterial hosts (Figge, 2006). A pathway for l-methionine biosynthesis was already successfully deregulated in *E.coli* (Huang *et al.*, 2017). In contrast to the studies that treat the regulatory restrictions, our example focuses on potential knockout strategies that reroute the metabolic flux and couple growth to a necessary overproduction of l-methionine.

We extended the *i*JO1366 model with the l-methionine proton antiporter *YjeH* (Liu *et al.*, 2015). In total we found 258 intervention strategies (MCSs) with a minimum of 9 and a maximum of 13 cuts. The MCSs can be grouped in 37 equivalence classes. From each class we picked the best representative as described above. Scoring and ranking was done as described in Section 2 using the weighting coefficients given in Section 3.2. The highest ranked candidate, MCS 41, consists of the 10 reaction deletions *TPI*, *ENO*, *CYSDS*, *GPDDA2*, *GPDDA2pp*, *HCYSMT*, *LSERDHr*, *MTHFC*, *SERD_L* and *TRPAS2* which can be established through the knockout of the genes *tpiA*, *eno*, *mmuM*, *folD*, *ydfG*, *sdaA*, *glpQ*, *sdaB*, *metC*, *ugpQ*, *tnaA* and *tdcG*.

**Fig. 3.**
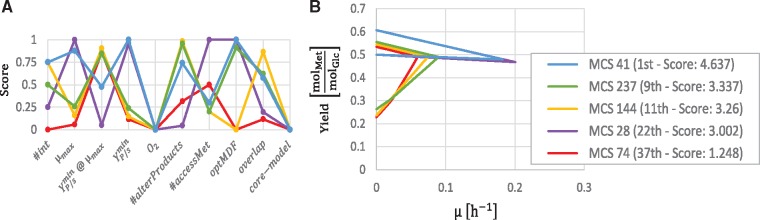
Comparison of five selected intervention strategies for the growth-coupled overproduction of l-methionine in *E.coli.***A**: Scores of the MCSs for the ten evaluation criteria. **B**: Growth-rate versus product-yield plot of the selected MCSs

Figure 3 shows the performance of five exemplary MCSs for the different criteria. As shown in Figure 3B, different MCSs can lead to growth-rate-product-yield (GRPY) spaces with similar shapes. Yet, the further assessment of the candidates reveals that a characterization solely on the basis of these trade-off plots is limited. MCSs with similar GRPY spaces can still perform very differently on other criteria, such as the thermodynamic driving force, the number of possible by-products or the number of necessary knockouts (Fig. 3A). For example, the highest ranked MCS (MCS 41 - blue) is more robust and needs less knockouts than MCS 28 (purple), even though their GRPY spaces are almost identical. In fact, it should be noted that there is only a small variance in the minimum product yields (at maximum growth rate) of all MCSs nevertheless leading to different scores for this criterion. As mentioned earlier, in those cases with low variability one could leave out the respective criterion/score by setting its weight to zero. While MCS 237 (green) is outperformed by MCS 144 (yellow) in the overlap score and the number of interventions, it is thermodynamically more favorable and offers a higher maximum growth rate. Figure 3B also shows that none of the five selected MCSs could be used in the *E.coli* core model *EcoliCore2* indicating that the computed ISs rely on pathways outside of the central metabolism with possibly lower capacities. A closer analysis revealed that the crucial pathway in this case starts with the glycine C-acetyltransferase reaction GLYAT that finally reintroduces glycine into the pyruvate metabolism. This pathway is not contained in the *EColiCore2* model.

**Fig. 4.**
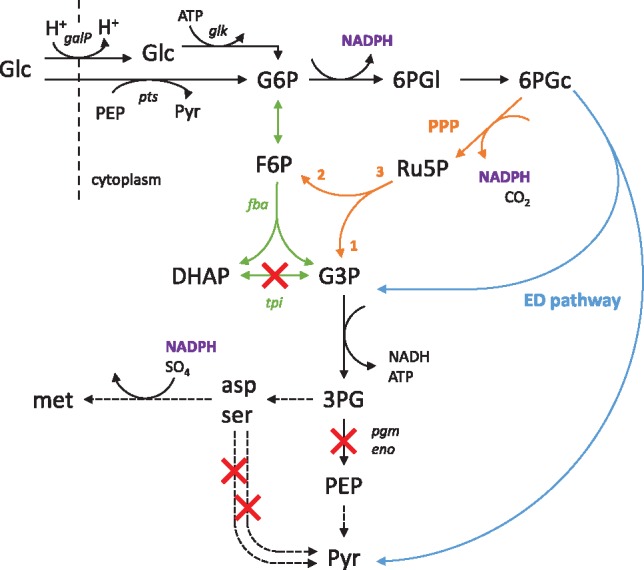
Strategies to couple growth with methionine synthesis in *E.coli* as revealed by the computed MCSs

We further analyzed the computed MCSs to understand the coupling mechanisms. In all MCSs the coupling was established through a combined deletion of the triose-phosphate isomerase reaction and one of the two final glycolysis steps catalyzed by phosphoglycerate mutase or enolase. As a result, sugar degradation has to take place along the Entner–Doudoroff pathway (ED) which forces the carbon flux to split into pyruvate and glyceraldehyde-3-phosphate (G3P) from where the metabolic pathway to PEP and pyruvate is blocked. The flux through pyruvate can join the TCA cycle to generate reduction equivalents mainly used for ATP synthesis via respiration. G3P cannot enter the TCA cycle directly and can only be drained via the pathways to serine and aspartate which are then further metabolized to methionine, consuming NADPH produced in the ED pathway (Fig. 4). In addition to *tpi* and *eno/pgm*, essential knockouts that occur in all MCSs are the reactions of the cysteine desulfhydrase (*CYSDS*), l-serine deaminase (*SERD_L*) and tryptophanase (*TRPAS2*) by which amino acid degradation pathways are blocked. While all MCSs contain the essential knockouts described above, specific interventions in the MCS now enforce different routes through the amino acid pathways with certain flux ratios. For the flux rerouting, different combinations of reaction knockouts are possible bearing different advantages and disadvantages. MCS 28 and 74 are less robust, as there are many more intermediates (e.g. up to 218 for MCS 28) which, if secreted, can abrogate growth coupling.

As described in Section 2 section (property 6), it was also tested, whether coupling is still existent when ATP is available at no cost, or electron sources or sinks are added that reduce or oxidize NAD(P)/H. We found that an artificial supply with ATP leads to the disruption of the growth coupling, suggesting that ATP synthesis (and therefore growth) is coupled to methionine synthesis. An external supply with electrons would also abolish coupling because the electron surplus can be used to increase ATP synthesis via respiration. Contrary, giving the mutants the option to drain electrons does not affect the coupling.

FBA and the thermodynamic OptMDF analysis predict the infeasibility of 134 out of 258 computed knockout strategies (19 out of 37 equivalence classes). These strategies rely on the reversed malate oxidase reaction (MOX), which is thermodynamically infeasible under the considered physiological conditions. Furthermore, in all strategies, the essential knockouts of the genes *tpiA* and *eno* or *pgm* represent serious interventions in the core metabolism of the cell. While the knockout of *tpi* has been proven to be feasible in *E.coli* (Facchetti, 2016), the additional knockouts of the enolase or phosphoglycerate mutase genes may be difficult targets. It was reported that the deletion of *pgm* leads to a mutant that could not grow on minimal medium (Foster *et al.*, 2010). A reason may be the limited flux capacity of the ED pathway in *E.coli* which could be overcome by additionally deregulating the ED pathway or by overexpressing its enzymes. Another potential drawback of the found intervention strategies is that the glucose uptake and activation relies on the proton symport (*galP*) and the glucokinase (*glk*) reaction because there would be not enough PEP available to use the PTS system (Hernández-Montalvo *et al.*, 2003). Hence, a key requirement for implementing the found strain design strategies is to enhance the capacity of the ED pathway in *E.coli*.

### 3.4 Example 2: 1,4-Butanediol as heterologous product

The metabolic engineering approach for the production of the non-natural bulk chemical 1,4-butanediol (BDO) by *E.coli* (Yim *et al.*, 2011) and its later commercialization has been an unprecedented success story for targeted metabolic engineering. In order to make this chemical producible by *E.coli*, Yim *et al.* designed a novel artificial pathway that branches from the tricarboxylic acid (TCA) cycle involving five heterologous enzymes. The strain design was supported by the computation of knockout strategies based on the OptKnock algorithm which suggested the repression of the main fermentation pathways and of the oxidative operation of the TCA cycle to couple growth with product synthesis through the intracellular redox balance. Later studies sought to further enhance the production performance of these strain designs through enzyme engineering (Hwang *et al.*, 2014) or extended kinetic modeling (Andreozzi *et al.*, 2016).

We integrated the pathway from 2-oxoglutarate to BDO, presented by Yim *et al.*, 2011, into the *i*JO1366 model (as well as in the *EColiCore2* model) and computed the minimal cut sets that establish strong growth coupling for BDO production with this pathway. We identified 274 MCS strategies with 6 to 12 reaction knockouts each, that could be grouped in 107 equivalence classes. We picked one representative per MCS class and ranked them as described before.

**Fig. 5.**
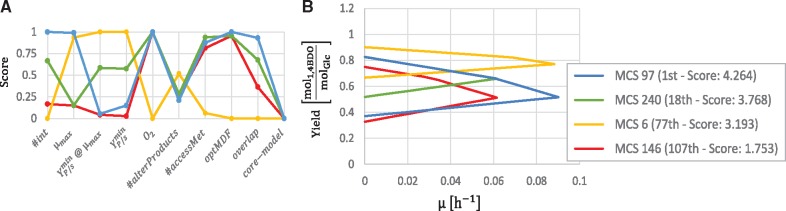
Comparison of four exemplary intervention strategies for the growth-coupled overproduction of 1,4-butanediol in *E.coli*. **A**: Scores of the MCSs for the different criteria. **B**: Growth-rate versus product yield plot of the four MCSs

In Figure 5, four MCSs are compared including the MCS with the highest and the lowest score. Figure 5A shows the single scores of the candidates among the different criteria, while Figure 5B shows their GRPY spaces. The best MCS (blue) does not have the highest minimum product yield, however, it allows anaerobic conditions and has a good balance between a high growth rate, a small number of necessary cuts (6), and an overlap with many other MCSs which would enable switching to another IS if necessary. Nevertheless, depending on the application, MCS 6 (yellow) might also be a relevant candidate. Even though it requires 12 cuts, it has a very good product yield and is more robust than the other MCSs. Sometimes, coupling can already be established with fewer interventions than the full set of a computed intervention strategy (see e.g. Harder *et al.*, 2016). Generally, the determination of the best candidate may have different outcomes. However, the ranking often shows intervention strategies that can be excluded a priori. For example, the worst strategies that (MCS 146, red) is outperformed by the best one (blue) in all criteria.

FBA and the thermodynamic OptMDF analysis predict the thermodynamic feasibility of all computed knockout strategies. In total, 104 out of 107 equivalence classes relied on anaerobic conditions all of which suggested the disruption of the tricarboxylic acid cycle (most frequently at malate dehydrogenase), the ethanol and lactate pathways, the knockout of the NAD(P) transhydrogenase (*THD2pp*) and then other (for each MCS specific) knockouts to establish full coupling. In all these strains, ATP synthesis is possible through glycolysis with the essential fermentation products formate, BDO and acetate. While acetate is a by-product of the BDO pathway, the ratio of formate and BDO is predetermined by the redox-state. Depending on the respective MCS, succinate and ethanol may occur as side products, ethanol as a product from a cycle in the glycine metabolism. The MCSs from the three remaining equivalence classes work under aerobic conditions and rely on the interruption of the TCA cycle through which BDO becomes an essential by-product of respiration. The intervention strategy pursued by Yim *et al.* (2011) relies on knockouts of the alcohol dehydrogenase (*adhE*), pyruvate formate lyase (*pfl*), lactate dehydrogenase (*ldh*) and the malate dehydrogenase (*mdh*) and has thus a large overlap with the computed MCSs. In addition, the aerobic respiration control protein (a*rcA*) was knocked-out by Yim *et al.* but not considered herein as it is a regulator protein and no metabolic enzyme. As predicted, acetate excretion was observed for these strains. Differences between the strategy of Yim *et al.* and the computed MCSs probably arise because (i) Yim *et al.* used a microaerobic process by which a knockout of the pyruvate formate lyase is allowed (this mutant cannot grow under strict anaerobic conditions) such that CO_2_ and acetate are the only essential by-products, and because (ii) the chosen knockouts of Yim *et al.* do actually not yet guarantee a BDO production in the *i*JO1366 model, even with simulated microaerobic conditions with an upper limit for oxygen uptake of 2 mmol/gDW/h.

The identified best MCS candidate (blue) consists of the reaction knockouts of the acetaldehyde dehydrogenase (*ACALD*), glucose 6-phosphate dehydrogenase (*G6PDH2r*), D-lactate dehydrogenase (*LDH_D*), malate dehydrogenase (*MDH*), phosphopentomutase 2 (*PPM2*) and the NAD(P) transhydrogenase (*THD2pp*). On the gene level this MCS can be established through the knockout of *mhpF*, *ldhA*, *pntB*, *zwf*, *mdh*, *adhE* and *deoB*. The list of all computed MCSs is provided in the Supplementary Material.

## 4 Discussion

Many constraint-based strain optimization methods can generate a pool of intervention strategies from which one candidate has to be selected for strain development. In this work we introduced methods for the characterization and ranking of such a pool of IS candidates with a focus on growth-coupled strain designs. We presented a catalogue of ten partially new criteria for assessing and ranking individual IS candidates, broadly extending earlier approaches such as OptPipe (Hartmann *et al.*, 2017) which assessed only four criteria (growth/production performance and an adaptability measure). Our ten criteria comprise (i) the number of interventions, (ii) the maximum growth rate, (iii) the minimum product yield at maximum growth rate, (iv) the overall minimum product yield, (v) the required aeration strategy, (vi) the number of alternative products that could disrupt growth coupling, (vii) the number of accessible (producible) metabolites, (viii) the maximal thermodynamic driving force, (ix) a score for the similarity to (overlap with) other ISs and (x) the feasibility of the IS in a reduced or further constrained model. Each criterion gives rise to a score which can be combined to an overall score useful to rank the IS pool. Even though the integration of all ranking criteria into the initial IS computation would be partially possible, in most cases it would be computationally too expensive rendering a posteriori ranking indispensable. Using real-world examples of strain design, we demonstrated the applicability and benefit of the developed characterization and ranking procedure. In our first example we computed and ranked sets of ISs for the strong coupling of growth with production of l-methionine in *E.coli*, while the second example focused on the production of 1,4-butanediol via a heterologous pathway. The case studies showed that, apart from the actual ranking, the analysis of an exhaustive set of ISs based on our ten criteria enables a thorough characterization of ISs, also supporting the elucidation of the underlying coupling mechanism. The comparison of ISs via the different criteria shows that the selection of the best candidate is often not trivial and involves trade-offs. Even though the highest ranked candidate usually performs well in multiple criteria (e.g. in the expected production performance) it may be outperformed in a subset of other criteria (e.g. in the robustness of the strain designs).

Depending on the specific needs, our ranking procedure could be easily adapted (e.g. by replacing our weighted score approach with the rank product method used by Hartmann *et al.*, 2017) or by analyzing additional properties. One example could be the number of undesired byproducts sometimes arising for certain ISs (e.g. acetate in the BDO case discussed above). Furthermore, a measure for the adaptability of the resulting mutant strains could be taken into account. For example, Hartmann *et al.*, 2017 used the MOMA method (Segrè *et al.*, 2002) to estimate the distance between the wild-type and the mutant flux distribution which can then be used as a criterion to rank ISs according to the required metabolic adjustments. However, we believe that this measure is not suited for a generic comparison of ISs for growth-coupled product synthesis, because it is the assumption of many strain design methods that the mutant strains evolve [via adaptive laboratory evolution (Conrad *et al.*, 2011)] towards growth-optimal phenotypes. Hence, phenotypes with minimal metabolic adjustment as predicted by MOMA will then not be relevant. Furthermore, MOMA and related methods require as input valid wild-type reference flux distributions which are often not known. This becomes even more critical if a series of intermediate strains needs to be constructed for which reference flux distributions are not available at the time of ranking the computed ISs.

As an important tool for characterizing and ranking strain designs, we also introduced the notion of equivalence classes of ISs by which ISs with identical solution spaces can be grouped. This simplifies the analysis as well as the ranking of the strategies since then only one representative for each class needs to be taken into account. Once the optimal strategy has been identified, possible alternative solutions in its equivalence class can be investigated.

The strength of our approach lies in its straightforward and generic applicability as it is independent from product, substrate, host organism and the computational method used to generate the IS candidates. The investigation of most criteria requires only an LP solver and the stoichiometric models of the wild type and the mutant. For the thermodynamic analysis, a relatively simple mixed-integer linear optimization problem must be solved (Hädicke *et al.*, 2018). We thus consider the proposed methodology a profound basis for the characterization and ranking of computed ISs with respect to their performance, robustness and implementation efforts which should help to speed-up the development of efficient strains for bio-based production processes.

## Supplementary Material

bty1065_Supplementary_File
